# Tissue Specific Deletion of Inhibitor of Kappa B Kinase 2 with OX40-Cre Reveals the Unanticipated Expression from the OX40 Locus in Skin Epidermis

**DOI:** 10.1371/journal.pone.0032193

**Published:** 2012-02-21

**Authors:** Georgina H. Cornish, Sim L. Tung, Daniel Marshall, Steve Ley, Benedict P. Seddon

**Affiliations:** 1 MRC National Institute for Medical Research, The Ridgeway, Mill Hill, London, United Kingdom; 2 King's College London, Academic Department of Rheumatology, Center for Molecular and Cellular Biology of Inflammation, New Hunt's House, Great Maze Pond, London, United Kingdom; University of Nebraska Medical Center, United States of America

## Abstract

NF-**κ**B signalling plays an essential role in T cell activation and generation of regulatory and memory populations *in vivo*. In the present study, we aimed to investigate the role of NF-**κ**B signalling in post-activation T cells using tissue specific ablation of inhibitor of kappa-B kinase 2 expression, an important component of the inhibitor of kappa-B kinase complex in canonical NF-**κ**B signalling. The OX40 antigen is expressed on activated T cells. Therefore, we used previously described mouse strain expressing Cre recombinase from the endogenous OX40 locus. Ablation of IKK2 expression using OX40^Cre^ mice resulted in the development of an inflammatory response in the skin epidermis causing wide spread skin lesions. The inflammatory response was characterised by extensive leukocytic infiltrate in skin tissue, hyperplasia of draining lymph nodes and widespread activation in the T cell compartment. Surprisingly, disease development did not depend on T cells but was rather associated with an unanticipated expression of *Cre* in skin epidermis, and activation of the T cell compartment did not require *Ikbk2* deletion in T cells. Employment of Cre reporter strains revealed extensive *Cre* activity in skin epidermis. Therefore, development of skin lesions was rather more likely explained by deletion of *Ikbk2* in skin keratinocytes in OX40^Cre^ mice.

## Introduction

The transcription factor NF- B regulates the expression of genes controlling cell survival, proliferation, immune stress responses and inflammatory reactions [1], [2], [3]. Canonical NF-**κ**B signalling is mediated by hetero- and homo-dimers of proteins belonging to the NF-**κ**B family. These dimers are held inactive in the cytoplasm by inhibitor of kappa B (I**κ**B) proteins. I**κ**Bs can be phosphorylated and ubiquitinated as a result of signalling induced by multiple stimuli including pro-inflammatory cytokines and antigen receptors particularly the T cell receptor (TCR). These modifications induce I**κ**B degradation via the proteosome and translocation of NF-**κ**B subunits into the nucleus for regulation of transcription [4]. Phosphorylation of I**κ**B is mediated by the I**κ**B kinase (IKK) complex, containing two catalytic subunits IKK1 (IKK**α**), IKK2 (IKK**β**) [5], [6] and a regulatory subunit, NF-**κ**B essential modulator (NEMO or IKK**γ**) [7], [8]. While IKK1 and IKK2 are highly homologous kinases, individual knockout mice produce different phenotypes; IKK1 deficient mice die shortly after birth with skeletal abnormalities and a thickened hyper proliferative epidermis [9], [10]. IKK2 knockout mice die at embryonic day 12.5–14.5 from a massive liver degeneration as a result of tumour necrosis factor (TNF)-induced hepatocyte apoptosis [11], [12], [13]. NEMO is a catalytically inactive NF-**κ**B modulator [7], [8], [14] and knockout mice exhibit male embryonic lethality due to massive hepatocyte apoptosis. In addition, heterozygote females develop patchy skin lesions with massive granulocyte infiltration, hyper proliferation and increased apoptosis of keratinocytes, reminiscent of incontinentia pigmenti (IP), which has subsequently become an animal model for this disease [15], [16].

In T cells of the immune system, NEMO activity is required for normal T cell activation. Perturbations to NF-**κ**B signalling in T cells either lacking the c-Rel subunit [17] or IKK2 [18], or in mice expressing a knock-in mutation of p105 that is resistant to IKK induced proteolysis [19], all result in impairment of TCR triggered proliferation. In some cases, this could be attributed to a failure to produce IL-2 [17], [19]. These defects in activation are also associated with defective T cell homeostasis, since these same strains all lack normal memory and regulatory T cell populations. However, whether this reflects a specific failure to either generate or maintain these populations remains unclear.

In the present study, we aimed to investigate the role of NF-**κ**B signalling in maintenance of memory and regulatory CD4 T cell populations by deleting IKK2 in mature T cells after their activation. This was achieved using a previously described *Cre* recombinase expressed from the endogenous OX40 gene locus (OX40^Cre^) [20]. OX40 is a member of the TNF receptor super family whose expression was thought to be restricted to CD4 T cells following their TCR dependent activation [21]. Surprisingly, deletion of a floxed *Ikbk2* allele with OX40^Cre^ resulted in a severe inflammatory phenotype characterised by development of skin lesions with extensive haematopoietic infiltrate and immune activation. Contrary to expectations, disease development did not depend on *Ikbk2* deletion in T cells, the anticipated site of OX40^Cre^ expression, but was rather associated with Cre activity in skin epidermis.

## Results

### Conditional deletion of *Ikbk2* using OX40^Cre^ driver results in severe skin pathology

**Figure 1:**
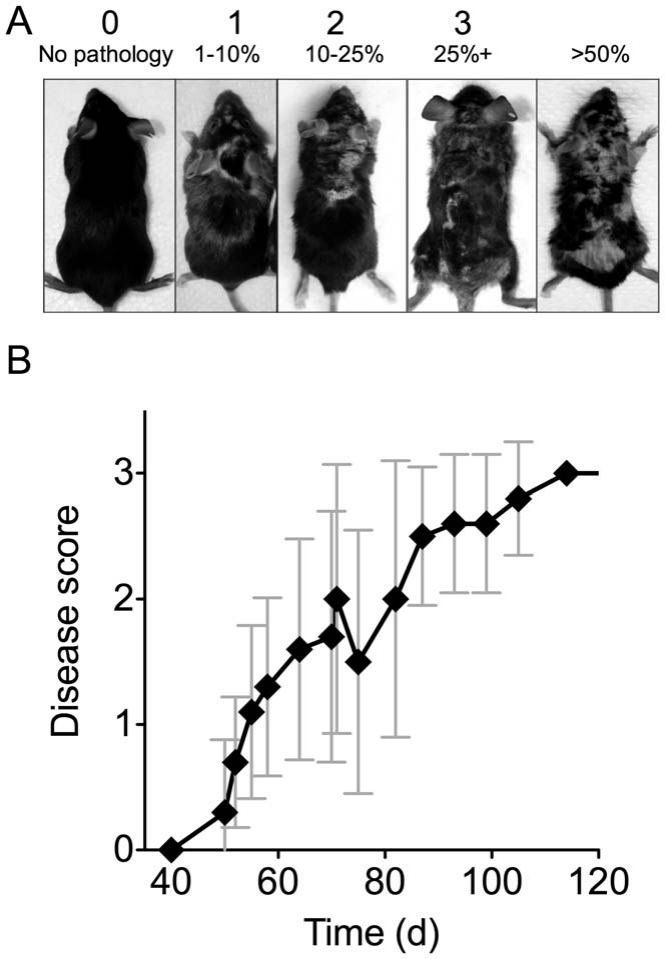
Development of skin pathology in *OX40^Cre^ Ikbk2^fx/fx^* mice. (A) Photographs show dorsal aspect of *OX40^Cre^ Ikbk2^fx/fx^* mice with varying degrees of skin pathology. Scoring was established according to extent of involvement of the dorsal surface: 0 - no pathology, 1 - <10% surface, 2 - 10–25% surface, 3 - >25% surface. Mice with >50% dorsal skin involvement and/or weight loss >20% were culled. (B) Graph shows average mouse score for *OX40^Cre^ Ikbk2^fx/fx^* mice over time (n = 9).

In order to investigate the role of NF-**κ**B activity in antigen experienced peripheral T lymphocyte populations *in vivo*, we took advantage of a previously described conditional allele of *Ikbk2* where exons 6 and 7 are flanked by two loxP sites (*Ikbk2^fx^* hereon) [22]. Conditional deletion of *Ikbk2* in T cells was achieved by generating homozygote *Ikbk2* ^fx/fx^ strain also carrying an insertion of the gene for *Cre* recombinase in the *Ox40* locus [20]. OX40 is a member of the TNF receptor family of proteins induced upon T cell activation [21] and also constitutively expressed on peripheral T_reg_ [23]. All mice were bred to express OX40^Cre^ in consideration of Cre toxicity [24] and *Ikbk2* ^fx/wt^ OX40^Cre^ mice, expressing a single WT *Ikbk2* allele, were bred as controls. Control *Ikbk2* ^fx/wt^ OX40^Cre^ mice were entirely healthy. Both strains were also homozygous for the Rosa26RYFP Cre reporter allele (See Materials and Methods). Unexpectedly, *Ikbk2* ^fx/fx^ OX40^Cre^ mice developed progressive skin lesions (Fig. 1A). Pathology was characterised by hair loss and a dry, flaky appearance. In order to grade disease severity, pathology was scored on the basis of the proportion of the dorsal surface affected (Fig. 1A, Methods). At late stages, mice with more than 50% involvement of the dorsal surface also exhibited weight loss and were therefore culled. Time course analysis of disease development revealed an onset of disease at approximately 5–6 weeks of age, followed by progressive degeneration of mouse health over subsequent weeks (Fig 1B).

### Epidermal hypertrophy and inflammation in *Ikbk2* ^fx/fx^ OX40^Cre^ mice

**Figure 2:**
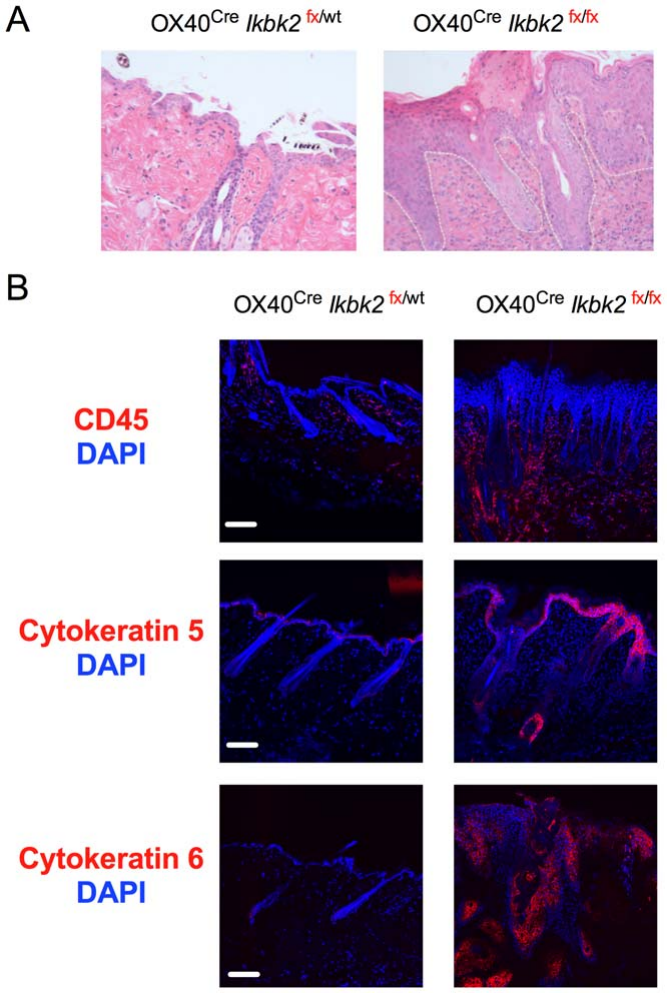
*OX40^Cre^ Ikbk2^fx/fx^* mice exhibit skin epidermis hyperplasia. (A) Images show skin sections from 12–16 wk old mice of the indicated strain stained with H&E and taken at 10× magnification. White dotted line indicates the border between dermis and epidermis. (B) Confocal images of skins sections from 12–16 week old mice of the indicated mice stained for expression of CD45, cytokeratin 5 or cytokeratin 6 (red) and counter stained with DAPI. White scale bar indicates 10 µm size. Data are representative of 3 independent experiments and at least two mice per strain experiment.

To investigate the basis for the skin pathology in *Ikbk2* ^fx/fx^ OX40^Cre^ mice, we first analysed skin tissue samples by histology. Skin sections from *Ikbk* ^fx/fx^ OX40^Cre^ mice and *Ikbk2* ^fx/wt^ OX40^Cre^ controls were stained with haematoxylin and eosin, revealing extensive epidermal hyperplasia and an increase in basal layer keratinocytes in the epidermis (Fig. 2A). *Ikbk2* ^fx/fx^ OX40^Cre^ mice also showed several changes reminiscent of human psoriasis, such as acanthosis, hyper- and parakeratosis as well as intraepithelial and intra-hair follicle formation of aggregates of neutrophils and macrophage (Fig. 2A).

We also analysed sections by confocal fluorescent microscopy. Staining sections for expression of CD45 confirmed the presence of an extensive leukocytic infiltrate in lesions (Fig. 2B). Cytokeratin 5 is highly expressed in mesothelium of basal layers in stratified epithelia, and is a marker for mesothelioma and squamous cell carcinoma [25], [26], [27], [28], [29]. Cytokeratin 6 is a lineage marker of keratinocyte differentiation and is expressed in normal hair follicles. Over expression of cytokeratin 6 in interfollicular epidermis correlates with invasive tumour and can mark remnants of destroyed hair follicles. Induction of cytokeratin 6 is also commonly seen in hyper proliferative skin disorders and in wound healing [30], [31]. Staining sections for expression of cytokeratins 5 and 6 revealed elevated levels of both proteins in skin sections from Ikbk2^fx/fx^ OX40^Cre^ mice (Fig. 2B). Cytokeratin 5 expression was much enhanced at the epidermis of *Ikbk2^fx^* ^/fx^ OX40^Cre^ mice and highly induced in hair follicles compared to *Ikbk2^fx^* ^/wt^ OX40^Cre^ controls. Expression of cytokeratin 6, a marker for interfollicular epidermal differentiation, was low in control hair follicles but highly induced in *Ikbk2^fx^* ^/fx^ OX40^Cre^ skin in both epidermis and several epithelial cell aggregates in the dermis. These aggregates may represent epidermal cysts that are remnants of destroyed hair follicles. Taken together, cytokeratin 5 and 6 expression is dramatically induced in skin lesions of *Ikbk2^fx^* ^/fx^ OX40^Cre^ mice suggesting conditions of hyper proliferation, loss of hair follicle integrity and the possibility of wound healing deficiencies in these mice.

### Increased T cell activation in *Ikbk2^fx^* ^/fx^ OX40^Cre^ mice

**Figure 3:**
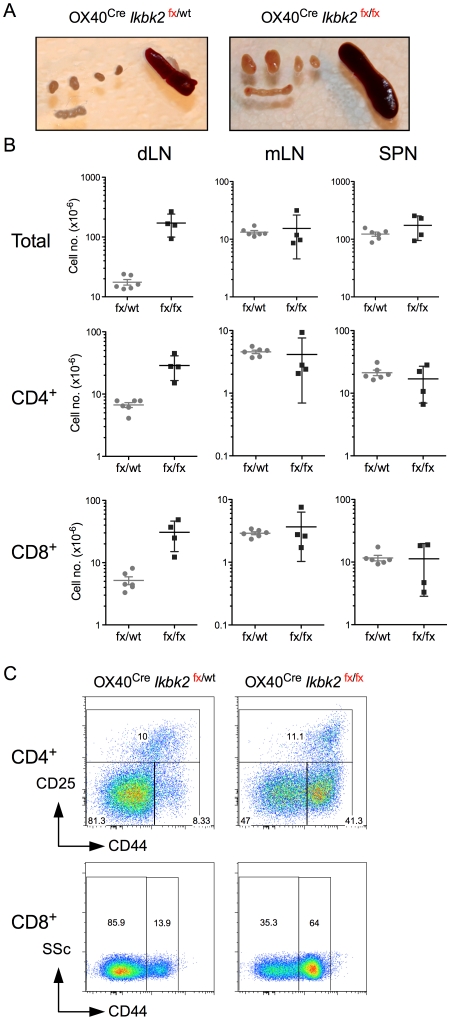
Lymphoid hyperplasia and T cell activation in *OX40^Cre^ Ikbk2^fx/fx^* mice. (A) Images show axillary and brachial lymph nodes (top four nodes) as compared with mesenteric chain and spleen from the indicated mouse strains, taken between 12–16 weeks of age. (B) Scatter charts show absolute numbers of total lymphocytes, CD4^+^ TCR^hi^ or CD8^+^ TCR^hi^ T cells in skin draining lymph nodes (dLN), mesenteric lymph nodes (mLN) or spleen (SPN) in *OX40^Cre^ Ikbk2^fx/fx^* mice (fx/fx, n = 6) or control *OX40^Cre^ Ikbk2^fx/wt^* mice (fx/wt, n = 4). (C) Density plots are of CD25 vs CD44 expression by CD4^+^ TCR^hi^ T cells (top row) and side scatter (SSC) vs CD44 by CD8^+^ TCR^hi^ T cells (bottom row) from dLN of the indicated mouse strains. Data are representative of six independent experiments.

Since we had initially generated *Ikbk2^fx^* ^/*fx*^ OX40^Cre^ mice in order to analyse IKK2 function in activated T cells, it was possible that the inflammatory skin condition was an autoimmune T cell response resulting from the *Ikbk2* deletion in T cells. We therefore examined the gross phenotype of the T cells in mice for evidence of perturbations to the homeostasis of this compartment. A more detailed functional analysis of *Ikbk2* deletion in T cells by OX40^Cre^ *in vivo* will be reported elsewhere (Cornish et al, manuscript in preparation). However, here we examined T cell numbers, phenotype and distribution in *Ikbk2^fx^* ^/*fx*^ OX40^Cre^ mice to look for evidence that T cells could be contributing to the observed skin pathology. Upon dissection of mice, it was immediately evident that lymph nodes draining the skin were enlarged in *Ikbk2^fx^* ^/*fx*^ OX40^Cre^ mice at 8 weeks of age (Fig. 3A). Therefore, analysis of lymph nodes was partitioned into those nodes that received afferent lymphatic drainage from skin, comprising cervical, brachial and axillary LNs (collectively dLN) and the mesenteric chain of lymph nodes (mLN) served by lymphatic vessels draining the gut. Confirming macroscopic observations, total cellularity of dLN recovered from *Ikbk2^fx^* ^/*fx*^ OX40^Cre^ was far greater when compared with *Ikbk2^fx^* ^/wt^ OX40^Cre^ controls. On average, mLN and spleen sizes were similar between strains (Fig. 3B). Counting CD4 and CD8 T cell numbers in organs also revealed their contribution to dLN hyperplasia in *Ikbk2^fx^* ^/*fx*^ OX40^Cre^ mice. Numbers of these T cell subsets in mLN and spleen remained similar to controls (Fig. 3B). We then analysed the composition of CD4 and CD8 T cells in dLN using CD44 and CD25 expression. CD4 T cells from *Ikbk2^fx^* ^/*fx*^ OX40^Cre^ mice had a substantial increase in the proportion of CD44^hi^ CD25^lo^ effector/memory phenotype cells, indicating greatly increased levels of immune activation in these mice. In contrast, proportions of CD25^hi^ cells, that are largely FoxP3^+^ T_reg_ but may also include recently activated effectors, were similar between strains. Similarly, CD8 T cells from *Ikbk2^fx^* ^/*fx*^ OX40^Cre^ mice also contained an enlarge subset of CD44^hi^ activated/memory phenotype cells, also suggestive of increased levels of immune activation.

### Development of skin pathology does not require T cells

**Figure 4:**
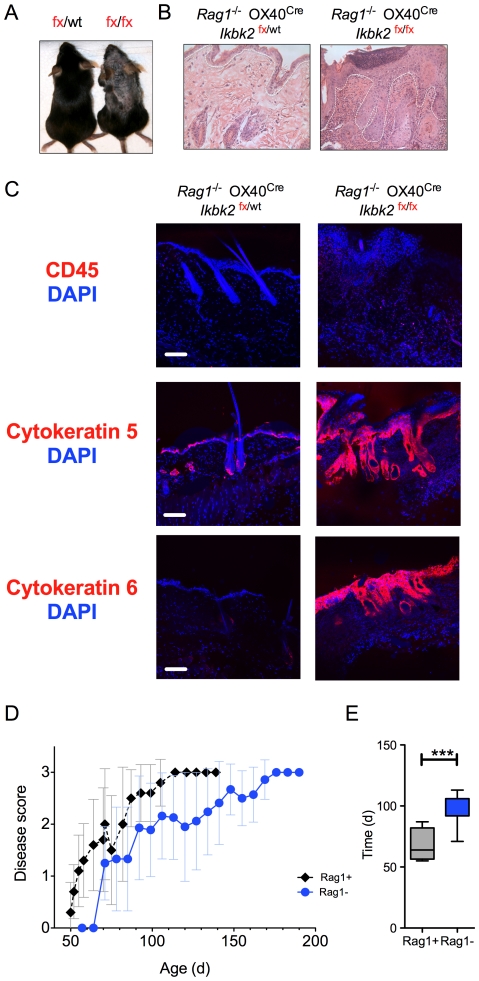
Skin pathology in *OX40^Cre^ Ikbk2^fx/fx^* mice is not T cell dependent. (A) Photograph shows skin pathology in a representative *Rag1* ^−/−^ *OX40^Cre^ Ikbk2^fx/fx^* mouse (fx/fx) compared with a littermate *Rag1* ^−/−^ *OX40^Cre^ Ikbk2^fx/wt^* control (fx/wt). White dotted line indicates the border between dermis and epidermis. (B) Images show skin sections from 12–16 week old mice of the indicated strain stained with H&E and taken at 10× magnification. (C) Confocal images of skins sections from 12–16 week old mice of the indicated mice stained for expression of CD45, cytokeratin 5 or cytokeratin 6 (red) and counter stained with DAPI. White scale bar indicates 10 µm size. (D) Graph shows progress of disease development in *Rag1* ^−/−^ *OX40^Cre^ Ikbk2^fx/fx^* mice (blue, n = 18) as compared with Rag1 sufficient *OX40^Cre^ Ikbk2^fx/fx^* mice (black lines, n = 9). (E) Bar chart shows mean time of disease progression to score 2 in *Rag1* ^−/−^ *OX40^Cre^ Ikbk2^fx/fx^* mice as compared with Rag1 sufficient *OX40^Cre^ Ikbk2^fx/fx^* mice. Data are representative (A–C) of three independent experiments or are pooled (D–E) from three independent experiments.

Although *Ikbk2^fx^* ^/*fx*^ OX40^Cre^ mice exhibited extensive leukocytic infiltration in skin lesions and evidence of increased immune activation in the T cell compartment, it remained unclear whether this was cause or effect of the on going skin pathology. To ask whether T cells were specifically required for development of skin pathology, *Ikbk2^fx^* ^/fx^ OX40^Cre^ mice were crossed onto a recombinase-activating gene 1 deficient (*Rag1*) background. Development of T lymphocytes in *Rag1* ^−/−^ mice is developmentally arrested in the thymus as cells cannot rearrange T cell receptor genes and therefore express mature TCR, essential for normal T cell development. Therefore, these mice have no mature T cells. Interestingly, *Rag1* ^−/−^ *Ikbk2^fx^* ^/*fx*^ OX40^Cre^ mice developed skin pathology that was very similar to their Rag1 expressing counterparts (Fig. 4A). Basic histological analysis revealed epithelial hyperplasia (Fig. 4B) similar to that described in Rag1 expressing mice (Fig. 2A). Similarly, confocal analysis of CD45, cytokeratin 5 and cytokeratin 6 expression levels revealed a comparable inflammatory phenotype to that described in the presence of T cells (Fig. 4C). Interestingly, analysing disease progression over time revealed that development of pathology in *Rag1* ^−/−^ *Ikbk2^fx^* ^/*fx*^ OX40^Cre^ mice was delayed, compared with Rag1 expressing *Ikbk2^fx^* ^/fx^ OX40^Cre^ mice (Fig. 4D). Mean time to reach disease score 2 was statistically significant between the two strains (Fig. 4E). Thus, T cells are not required for the development of the skin pathology in *Ikbk2^fx/fx^* OX40^Cre^ but there was evidence that their presence could exacerbate the course of pathology.

### OX40-Cre targets deletion of IKK2 in skin epithelia

**Figure 5:**
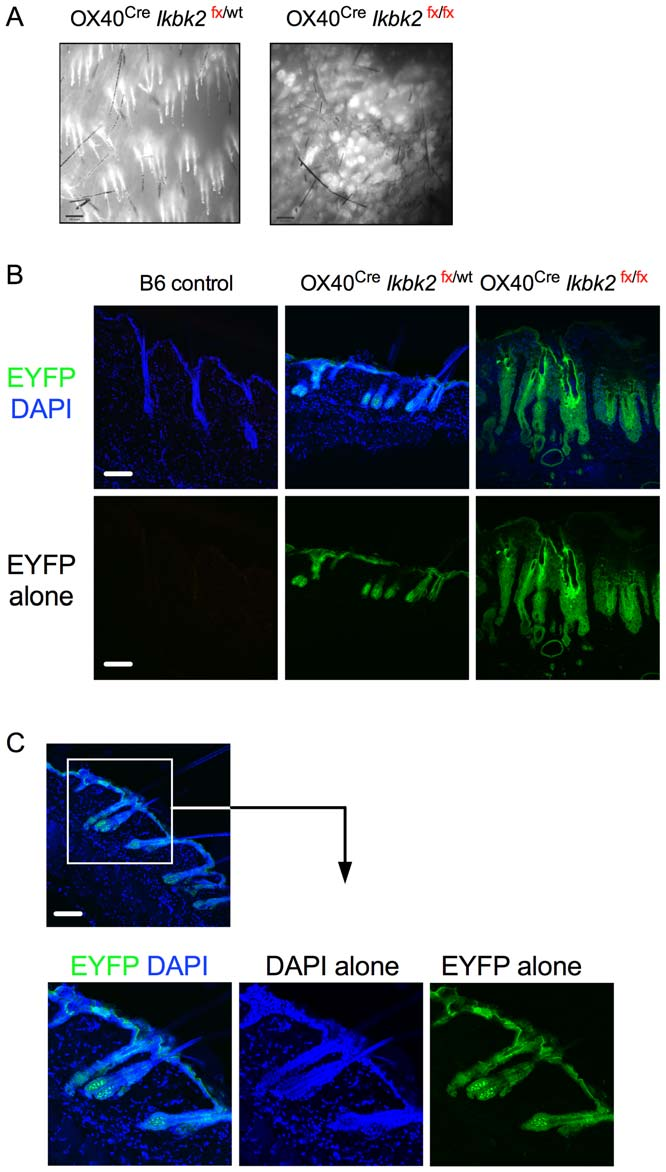
*Cre* EYFP reporter expression in the skin of OX40^Cre^ strains. (A) Fluorescent stereomicroscope images of epidermal skin layer taken from the underside (×10 magnification). (B) Confocal images of skins sections from 12–16 week old mice of the indicated mice showing expression of EYFP and counter stained with DAPI. (C) EYFP expression in hair follicle of healthy *OX40^Cre^ Ikbk2^fx/wt^* mice showing DAPI and EYFP separately and together. White scale bar indicates 10 µm size. Data are representative of five or more mice from three independent experiments.

The anticipated target tissue for the activity of OX40^Cre^ was in activated and regulatory CD4^+^ T cells. However, development of skin pathology in *Rag1* ^−/−^ *Ikbk2^fx/fx^* OX40^Cre^ mice lacking T cells suggested that pathology induced by OX40^Cre^ activity must be mediated by gene deletion outside of the T cell compartment. We therefore asked whether OX40^Cre^ was active within the skin tissue itself, and whether this could account for the development of disease in these mice. To facilitate this analysis, *Ikbk2^fx/fx^* OX40^Cre^ mice were additionally bred with a Rosa26R^EYFP^ *Cre* reporter mouse. This strain carries a modified Rosa26 locus containing the YFP gene with upstream trimeric TpA stop sequences flanked by loxP recombination sites (R26R^EYFP^) [32]. Cells that express *Cre* will excise the TpA stop sequences thereby releasing YFP expression, which persists both after cessation of *Cre* expression and in any cellular progeny. We initially analysed YFP expression in skin by stereo fluorescent microscopy (Fig. 5A). Both control *Ikbk2^fx/wt^* OX40^Cre^ R26R^EYFP^ and *Ikbk2^fx/fx^* OX40^Cre^ R26R^EYFP^ strains exhibited dense EYFP expression around hair follicles in the skin. We then examined EYFP expression by confocal fluorescent microscopy to more precisely determine the cellular localisation of EYFP expression. In control *Ikbk2^fx/wt^* OX40^Cre^ R26R^EYFP^ mice a thin layer of EYFP expression was localized to the epidermis (Fig. 5B). Analysis of skin from *Ikbk2* ^fx/fx^ OX40^Cre^ R26R^EYFP^ mice also showed eYFP expression in the epidermis that was more extensive than in controls (Fig. 5B), consistent with the enlarged epidermis in these mice (Fig. 2A). Interestingly, more detailed analysis of healthy control *Ikbk2^fx/wt^* OX40^Cre^ R26R^EYFP^ mice revealed that EYFP expression extended into hair follicles, and was most brightly expressed in cellular structures in the bulb of the hair follicle. Since EYFP expression does not necessarily report current expression of OX40^Cre^, we also attempted to directly assess expression of OX40 in skin by immunostaining. Staining could not reveal OX40 antigen expression in any samples (data not shown). However, this may represent a limit on detection with the mAb used, rather than a genuine absence of OX40 protein expression.

### Immune activation in *Ikbk2* ^fx/fx^ OX40^Cre^ mice is independent of IKK2 deletion in T cells

**Figure 6:**
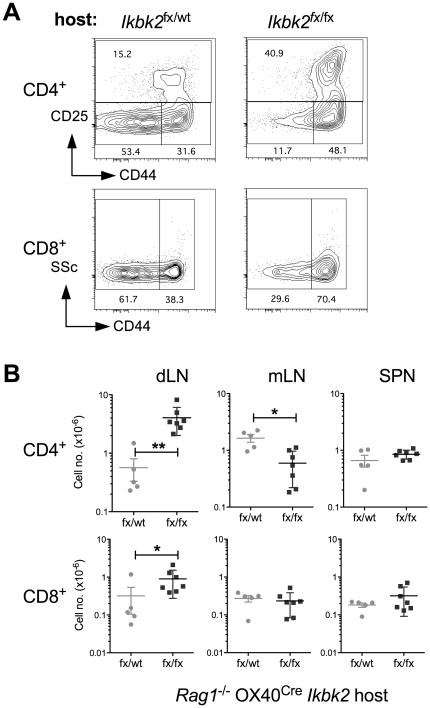
Activation of WT T cells in *Rag1* ^−/−^ *OX40^Cre^ Ikbk2^fx/fx^* mice. Eight week old *Rag1* ^−/−^ *OX40^Cre^ Ikbk2^fx/fx^* and *Rag1* ^−/−^ *OX40^Cre^ Ikbk2^fx/wt^* controls were reconstituted with 5×10^7^ lymph node T cells from WT C57Bl6^NIMR^ mice. Two weeks later, number and phenotype of transferred T cells in different recipients was determined. (A) Contour plots are of CD25 vs CD44 expression by CD4^+^ TCR^hi^ T cells (top row) and side scatter (SSC) vs CD44 by CD8^+^ TCR^hi^ T cells (bottom row) from dLN of *Rag1* ^−/−^ *OX40^Cre^ Ikbk2^fx/fx^* (*Ikbk2* fx/fx) and *Rag1* ^−/−^ *OX40^Cre^ Ikbk2^fx/wt^* (*Ikbk2* fx/wt) hosts. Numbers indicate % of cells in the adjacent gate. Data are representative of at least five mice per strain. (B) Scatter charts show absolute numbers of CD4^+^ TCR^hi^ or CD8^+^ TCR^hi^ T cells in skin draining lymph nodes (dLN), mesenteric lymph nodes (mLN) or spleen (SPN) in host *Rag1^−/−^ OX40^Cre^ Ikbk2^fx/fx^* mice (fx/fx) or control *Rag1^−/−^ OX40^Cre^ Ikbk2^fx/wt^* mice (fx/wt). Data are pool of two independent experiments.

The presence of EYFP expression in skin epidermis demonstrated that OX40^Cre^ is active in skin epithelia, as well as in activated T cells as previously reported [20]. Since development of skin pathology occurred in the absence of T cells in *Rag1* ^−/−^ *Ikbk2^fx/fx^* OX40^Cre^ mice, we also questioned whether the immune activation observed in the T cells of *Ikbk2^fx/fx^* OX40^Cre^ mice was dependent on *Ikbk2* deletion in the T cell compartment or was rather a secondary phenotype induced by the on going pathological processes in the skin. Evidence for the latter view already comes from the observation that dLN in *Ikbk2* ^fx/fx^ OX40^Cre^ mice were specifically enlarged, implying that immune activation was triggered by processes in the skin rather than being a generalised phenotype throughout the T cell compartment of these mice. However, to directly test whether immune activation depended on OX40^Cre^ mediated IKK2 deletion in T cells, we examined the behaviour of WT T cells transferred to either *Rag1* ^−/−^ *Ikbk2* ^fx/fx^ OX40^Cre^ or healthy *Rag1* ^−/−^ *Ikbk2* ^fx/wt^ OX40^Cre^ control mice. Because these strains are T cell deficient and therefore profoundly lymphopenic, we adoptively transferred high doses of T cells into recipients to minimise induction of homeostatic proliferative responses that can also result in generation of CD44^hi^ activated/memory phenotype cells [33]. Two weeks after cell transfer, dLN, mLN and spleen were recovered from recipient mice and analysed by FACS. Both CD4^+^ and CD8^+^ WT donor T cells from dLN of *Rag1* ^−/−^ *Ikbk2* ^fx/fx^ OX40^Cre^ mice exhibited signs of immune activation compared with the same WT donor T cells transferred to healthy *Rag1* ^−/−^ *Ikbk2* ^fx/wt^ OX40^Cre^ mice. There was a substantial increase in proportion CD44^hi^ activated/memory phenotype cells in both CD4 and CD8 compartments (Fig. 6A). Interestingly, CD4^+^ CD25^+^ cells were also increased in representation amongst WT donor T cells recovered from *Rag1* ^−/−^ *Ikbk2* ^fx/fx^ OX40^Cre^ mice. Calculating absolute numbers of CD4 and CD8 T cells in different lymphoid organs revealed a specific accumulation of both subsets in dLN but not mLN or spleen from *Rag1* ^−/−^ *Ikbk2* ^fx/fx^ OX40^Cre^ mice, resembling the dLN hyperplasia observed in intact *Ikbk2* ^fx/fx^ OX40^Cre^ mice (Fig. 3). Therefore, the immune activation observed in *Ikbk2* ^fx/fx^ OX40^Cre^ appears to be a characteristic intrinsic to the host environment in *Ikbk2* ^fx/fx^ OX40^Cre^ mice and not the T cell compartment, suggesting that the skin pathology is responsible for inducing the observed T cell response.

### Skin pathology is associated with TNF-α production in skin of *Ikbk2* ^fx/fx^ OX40^Cre^ mice

**Figure 7:**
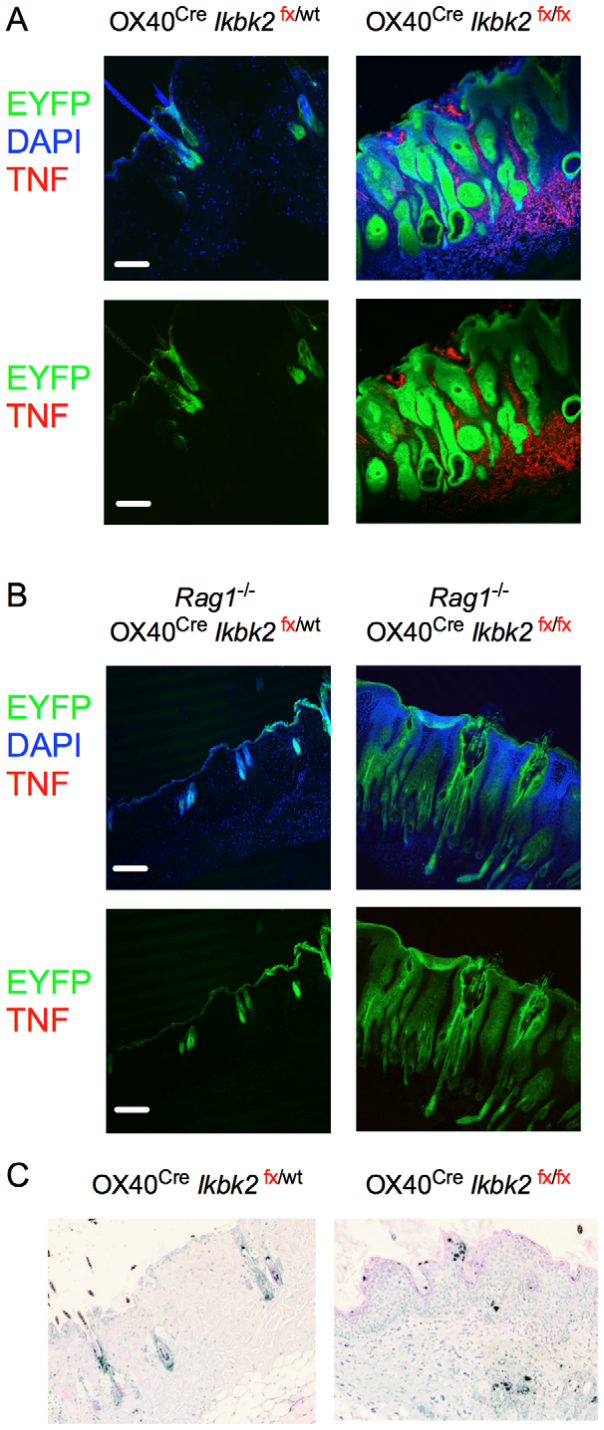
Skin pathology in *OX40^Cre^ Ikbk2^fx/fx^* mice is associated with TNF production and increased apoptosis. (A) Confocal images of skins sections from 12–16 week old mice of the indicated strain were analysed for EYFP expression (green), stained with DAPI (blue) and for expression of TNF (red). White scale bar indicates 10 µm size. (B) Apoptosis was assessed in skin sections from *OX40^Cre^ Ikbk2^fx/fx^ and OX40^Cre^ Ikbk2^fx/wt^* mice by TUNNEL assay and counterstained with eosin. Light microscopy was performed at 10× magnification.

The skin pathology we report here closely resembles that reported in mice where *Ikbk2* is conditionally deleted in skin keratinocytes using the constitutive K14^Cre^ and inducible K14^CreERT2^ driver strains [31], [34]. From these studies it can be concluded that adult keratinocytes deficient in IKK2 aberrantly produce inflammatory mediators driving skin pathology, independently of developmental processes in the skin. This skin pathology was hallmarked by epidermal hyperplasia, enhanced TNF expression and increased cell death. The role of TNF in pathophysiology of disease was illustrated in TNF receptor deficient mice, in which the skin pathology resolves. These studies propose that keratinocytes are sensitive to TNF induced death in the absence of IKK2 dependent pro-survival signals. To see whether a similar mechanism could account for the pathology we observed in *Ikbk2* ^fx/fx^ OX40^Cre^ mice, we analysed skin samples for evidence of elevated TNF production and apoptosis. Significantly, analysing skin samples by confocal fluorescent microscopy revealed extensive TNF production throughout the dermis of *Ikbk2* ^fx/fx^ OX40^Cre^ mice not observed in controls (Fig. 7A). We also analysed *Rag1* ^−/−^ *Ikbk2* ^fx/fx^ OX40^Cre^ mice for TNF expression. Surprisingly, TNF levels were much reduced in *Rag1* ^−/−^ mice (Fig. 7B). The difference in TNF levels also correlated with rate of disease onset, which was more rapid in *Ikbk2* ^fx/fx^ OX40^Cre^ mice than Rag1 deficient counterparts (Figure 4D). We then assessed the level of apoptosis in skin sections by TUNNEL assay. Significantly, examining skin from *Ikbk2* ^fx/fx^ OX40^Cre^ mice revealed evidence of high levels of apoptosis throughout the dermis and epidermis (Fig. 7C).

## Discussion

In the present study, we employed an OX40^Cre^ driver mouse line in order to delete IKK2 in T cells post activation. Unexpectedly, mice developed a severe and progressive inflammatory skin condition characterised by epidermal hyperplasia, leukocytic infiltrate and extensive T cell activation in draining lymph nodes. Although pathology superficially resembled an autoimmune response, disease was found not to depend on either T cells or deletion of the *Ikbk2* gene in activated T cells, but rather the unforeseen deletion of IKK2 in skin epithelia by the OX40^Cre^ driver.

The role of NF-**κ**B signalling in skin epithelial homeostasis has previously been investigated by tissue specific deletion of *Ikbk2* using K14-Cre driver [31], [34]. These mice developed severe skin pathology similar to that observed in the present study. In both cases, skin inflammation was associated with extensive leukocytic infiltration; epithelial hyperplasia enhanced production of TNF and increased levels of apoptosis. The K14 promoter drives *Cre* expression in keratinocytes within skin epithelium. Using R26RYFP reporter strain mice allowed us to map the extent of Cre activity in OX40^Cre^ mice. We found extensive YFP expression throughout the skin epithelia, and we would therefore anticipate similarly extensive deletion of *Ikbk2* in the same tissue. The development of skin pathology comparable to that observed in K14-Cre *Ikbk2* ^fx/fx^ mice suggests that there was extensive deletion of IKK2 in these same cells. It remains unclear however whether OX40^Cre^ is expressed constitutively in skin epithelial cells or whether it is expressed only at a specific developmental stage. In this regard, the extensive YFP expression in hair follicles may be significant, since this is also thought to be the site of origin of stems cells for skin epithelia [35], [36], [37].

Attempts to detect OX40 protein expression in skin were unsuccessful. It is possible that the antibody reagents were not sufficiently sensitive to detect the levels of OX40 potentially expressed in skin. Alternatively, OX40 expression may rather be limited to transient expression in a rare epithelial stem cell precursor population. In future studies, it would be interesting to compare the pattern of Cre activity between OX40^Cre^ and K14^Cre^, since K14 expression may be restricted to more differentiated stages of keratinocyte development, and OX40^Cre^ may represent a tool to target skin epithelia progenitors. However, until these studies are performed, this remains a point of speculation. It is also a formal possibility that introduction of the Cre cDNA, a bacterial gene, to the endogenous OX40 locus caused the ectopic expression of the OX40 locus in skin epithelial tissues. Until endogenous OX40 gene expression is detected in skin epithelial cells, this possibility cannot be ruled out. Previous studies have detected OX40 transcription in a wide range of tissues, including lung, skeletal and heart muscle, and testis [38], while others report expression in leukocytes other than T cells, such as NK cells. Consistent with this, a small (<1%) but detectable population of YFP^+^ TCR^−^ leukocytes was apparent in lymphoid tissues (Cornish et al, in preparation). We could confirm that OX40^Cre^ was active in the male germline, as offspring of OX40^Cre^ males frequently resulted in non-specific activation of the YFP reporter in the absence of OX40^Cre^ allele in offspring, as reported previously [20]. While we cannot exclude the possibility that introduction of the Cre gene to the endogenous OX40 locus has in some way perturbed normal gene expression resulting in ectopic expression in skin epithelia, we can confirm that OX40^Cre^ does faithfully report OX40 activity at least in T cells and testis as previously reported. The activity of OX40^Cre^ within skin epithelia may be relevant for both past [39], [40], [41], [42] and future studies using this driver to induce tissue specific gene deletion, especially in the light of the impact of dysregulated skin homeostasis on the normal T cell compartment. Whether OX40^Cre^ drives deletion in other tissues reported to express OX40 remains to be determined. The potential physiological role of OX40 gene activity in skin epithelium is unclear. However, there are no reports of skin abnormalities in OX40 deficient mice, so it does not appear that there is a non-redundant role for OX40 in the homeostasis of normal skin epithelia. Never the less, the OX40^Cre^ line employed in the present study mediated very efficient deletion of loxP-flanked genes in skin epidermis.

Our study also demonstrates how inflammatory processes can result in profound activation of the adaptive immune system, even in the apparent absence of foreign antigen. Our original aim had been to study the role of IKK2 signalling in activated T cells using *Ikbk2* ^fx/fx^ OX40^Cre^ mice. Therefore, when we observed the development of skin inflammation that was associated with substantial local activation of T cells in draining lymph nodes, it was natural to suppose that this was a consequence of the T cell specific deletion of *Ikbk2*. However, we found this was not the case. Skin pathology did not depend on the presence of T cells in *Rag1* ^−/−^ *Ikbk2* ^fx/fx^ OX40^Cre^ mice, although disease development was significantly slower in this strain. Furthermore, immune activation similar to that in intact *Ikbk2* ^fx/fx^ OX40^Cre^ mice was observed when *Rag1* ^−/−^ *Ikbk2* ^fx/fx^ OX40^Cre^ mice were reconstituted with WT T cells, so did not require *Ikbk2* deletion in T cells. Indeed, the resulting skin condition was rather exacerbated by the transfer of WT T cells for just 2 weeks (data not shown) compared with intact *Ikbk2* ^fx/fx^ OX40^Cre^ mice. This observation is also consistent with the elevated levels of TNF detected in *Ikbk2* ^fx/fx^ OX40^Cre^ as compared with the *Rag1* ^−/−^ *Ikbk2* ^fx/fx^ OX40^Cre^ strain. The presence of T cells in mice clearly exacerbated TNF production in the skin. This could either have been a result of direct production by T cells or an indirect consequence of T cells on inflammatory cells such as macrophages. Previous studies demonstrated the importance of TNF for pathology by rescuing mice from pathology by the introduction of Tnfr1 deficiency [34]. Future studies could use a similar strategy to confirm the importance of TNF signalling in *Rag1* ^−/−^ *Ikbk2* ^fx/fx^ OX40^Cre^ mice, especially as TNF levels were clearly lower in these mice than the T cell sufficient strain. In other studies, haematopoietic reconstitution of WT mice with bone marrow from *Ikbk2* ^fx/fx^ OX40^Cre^ donors resulted in normal T cell reconstitution and recipients showed no sign of the skin inflammation observed in intact *Ikbk2* ^fx/fx^ OX40^Cre^ donors (Cornish et al, in preparation). Thus, restricting *Ikbk2* deletion to the haematopoietic system is not sufficient to induce skin pathology. Furthermore, the activation within the T cell compartment of *Ikbk2* ^fx/fx^ OX40^Cre^ mice does not depend on *Ikbk2* deletion in T cells but appears to be secondary to the on going inflammatory process in the skin.

It remains to be determined what is driving the extensive immune activation observed in *Ikbk2* ^fx/fx^ OX40^Cre^ mice. The enlargement of draining lymph nodes is likely to be in part due to increased trafficking of cells into lymph nodes in response to inflammatory mediators, which are known to increase permeability of high endothelial venules that serve lymph nodes. However, such recruitment cannot account for the substantial increase in CD44^hi^ activated/memory phenotype cells in both CD4 and CD8 subsets that must in part be the result of activation and expansion of naïve precursors. It remains to be determined whether the expansion is antigen specific or the result of altered T cell homeostasis secondary to the effects of inflammatory mediators on dendritic and stromal cell components of the lymph node that are sources of IL-15 and IL-7 respectively [43], [44], [45] required for memory cell homeostasis. If the response is antigen specific, it will be interesting to investigate whether activation is in response to foreign antigen, such as environmental bacteria that have infected following the breakdown of skin epithelial barrier, or whether the response to altered self presentation by antigen presenting cells trafficking from the inflamed tissue. The answers to these questions could have important implications for understanding how the T cell compartment as a whole reacts to inflammatory processes.

## Materials and Methods

### Mice

Mice used in this study were: *Ikbk2^fx^* ^/fx^ [22], OX40^cre^ recombinase [20]; Rosa26RYFP [32], C57Bl6/J.*Rag1* ^−/−^, C57Bl/6^NIMR^. OX40^Cre^ transmission was restricted to female mice as OX40 is expressed in testis [20]. All *Ikbk2^fx^* OX40^Cre^ strains used in this study were also homozygous for the Rosa26RYFP allele. Mice were bred and maintained at the National Institute for Medical Research. Mice were bred and maintained at the MRC National Institute for Medical Research. Experiments were performed according to U.K. Home Office regulations. The study was conducted under Project License 80/2092 that has been approved by both the NIMR Ethical Review Panel and the U.K. Home Office.

### Pathology

Microscopic pathological assessment of embedded skin sections was conducted by Dr Madhuri Warren, Pathology Diagnostics, Cambridge, U.K. Whole live mouse pathology scoring was carried out at the NIMR under the following criteria, assessing the level of involvement of the dorsal surface; 0 = no hair loss; 1 = 1–10% hair loss; 2 = 10–25% hair loss and may include dermatitis and itching, 3>25% hair loss and may include dermatitis, itching, reddening skin, reduced weight. An endpoint was defined as meeting any of the following criteria; >50% hair loss, any signs of weakness or distress, or weight loss >20% starting.

### Immunofluorescence

Mice were shaved free of long hair, skin harvested, fixed in 4% paraformaldehyde for 2 hr and transferred into 30% sucrose rotating overnight at 4°C. Samples were then embedded in OCT compound and frozen. 25 mm cryostat sections were cut and mounted on slides for immuno-staining. The cryostat sections were left to air dry for 1 hour in the dark then transferred to −20°C freezer until staining. Frozen sections were taken from the freezer and left at room temp for 2 mins, incubated in PBS for 5 mins and permeabilized with 0.1% triton-X for 30 min at room temp. Slides were incubated firstly with mouse serum for 30 mins at room temp in a humid chamber and then purified antibodies re-suspended in PBS for 1 hr at RT at the following concentrations: Rabbit anti-mouse CD45 (14-0451-82, eBioscience) 1/50; Rabbit anti-mouse Cytokeratin 5 (ab24647-50, Abcam) 1/200; Rabbit anti-mouse Cytokeratin 6 (ab24646-50, Abcam) 1/200; Rabbit anti-mouse TNF-alpha (14-7321-85, eBioscience) 1/20. Rabbit antibodies were detected with goat anti-rabbit IgG AF546 (A-11010 10 µg/mL Molecular Probes) or Goat anti-rat IgG (H+L) AF647 (A21247 10 µg/mL Molecular Probes) for 1 hr in a dark humid chamber at RT re-suspended in PBS. All slides were counter stained with 1.5 ug/ml DAPI vectashield mounting medium (H-1200 Vector Laboratories). Apoptosis was detected in paraffin embedded skin sections using DERMATacs tunnel staining kit (Trevigen) as per manufacturers instructions.

### Flow cytometry

Phycoerythrin (PE)-conjugated monoclonal antibody (mAb) against CD8; Texas Red (TR)-conjugated mAb against CD4; PE-Cy5-conjugated mAb against TCRβ; PE-Cy7-conjugated mAb against CD25; Pacific Blue (PB)-conjugated mAb against CD44. Flow cytometry was performed with 2×10^6^ lymph node cells and splenocytes harvested and disassociated in air-buffered IMDM 10% FCS at 4°C. Cell concentrations were determined by a Scharf Instruments Casy Counter. Cells were incubated with saturating concentrations of antibodies in 100 µl of phosphate-buffered saline (PBS), 0.1% bovine serum albumin (BSA), 1 mM sodium azide (PBS-BSA-azide) for 1 hour at 4°C followed by one wash in PBS-BSA-azide. Seven-colour cytometric staining was analysed on CyAn instruments (Dako), and data analysis and colour compensation were performed with FlowJo V9 software (TreeStar). The data are presented on log and biexponential displays.
